# The influence of athletic performance on the highest positions of the final ranking during 2017/2018 Serie A season

**DOI:** 10.1186/s13102-021-00259-3

**Published:** 2021-03-25

**Authors:** Umile Giuseppe Longo, Francesco Sofi, Vincenzo Candela, Laura Risi Ambrogioni, Giuditta Pagliai, Carlo Massaroni, Emiliano Schena, Matteo Cimmino, Fabrizio D’Ancona, Vincenzo Denaro

**Affiliations:** 1 Department of Orthopaedic and Trauma Surgery, Campus Bio-Medico University, Via Alvaro del Portillo, 200, Trigoria,, 00128 Rome, Italy; 2 Centro Integrato di Ricerca (CIR), Campus Bio-Medico University, Rome, Italy; 3 Department of Experimental and Clinical Medicine, University of Florence, Florence, Italy; 4 Don Carlo Gnocchi Foundation, Onlus IRCCS, Florence, Italy; 5 Unit of Measurements and Biomedical Instrumentation, Department of Engineering, Campus Bio-Medico University, Rome, Italy

**Keywords:** Soccer, Football, Performance analysis, Serie A, Match statistics, Elite football, Sport

## Abstract

**Background:**

Our previous study on the 2016/2017 Serie A season showed that a greater likelihood of reaching the top positions in the Italian league “Serie A” seemed to be mainly related to sprint activity, goal attempts, total throws, target shots and assists. Therefore, we aim to evaluate the following season data in the same league to compare, confirm, and improve these results.

**Methods:**

The data of all the matches played during the “Serie A” 2017–2018 season were obtained from the Italian soccer league. The teams’ analysis was performed in terms of total distance covered in km, jogging, running and sprint activities, average speed, and match statistics (total shots, shots on target, goal attempts, assists, turnovers, and steals).

**Results:**

Teams that reached the first four positions revealed a lower percentage of running (65.98 ± 1.51 vs. 66.84 ± 2.18; *p* < 0.001), a higher percentage of jogging (25.61 ± 1.71 vs. 25.30 ± 1.97; *p* = 0.037) and sprint activities (8.41 ± 1.04 vs. 7.86 ± 0.82; *p* < 0.001). Match statistics seem to be statistically different between the first four teams the other teams. The total goals are strongly associated both with the total score at the end of the championship (*R* = 0.906; *p* < 0.001) and with the position in the final ranking (*R* = 0.850; *p* < 0.001).

**Conclusions:**

Our results suggest that high-level teams have a lower running rate and a higher percentage of jogging and sprinting than other teams.

## Background

Soccer is the most popular sport worldwide, both at the amateur and professional level [1]. It is a multi-skilled team sport that requires psychological, tactical, technical, and physical factors throughout the game [2–8]. Professional teams participating in professional competitions must have players in the best physical and psychological condition [9, 10]. Therefore, adequate physical preparation of elite players has become a mandatory part of professional soccer training to face the sport’s physical challenges [11, 12].

Cinematic analysis of players during the game has been implemented since it can provide helpful information about their performance to design optimal match strategies [13, 14]. For example, when walking, jogging, and running at different intensities and directions, the distance covered by players can be used to evaluate player performance during competitions and plan more efficient training sessions [15].

Previous studies that analyse players’ performance during the game [15–17] and at different periods of the match [6, 18] have observed that the athletic performance of the teams may depend on high intensity running, fatigue, sprint characteristics, distance covered, and the number of goals and actions. Although several studies have identified which variables might influence athletic performances, by far, there are no studies that statistically evaluate how these variables may affect the possibility of achieving higher positions in the final ranking.

Almost every country has its league for professional soccer teams. The Serie A is the highest professional level in the Italian football league, and it ranks third in the UEFA standings after the Spanish Liga and the English Premier League. A previous study on 2016/2017 “Serie A” season showed that a higher likelihood of reaching the top positions seemed to be mainly related to sprint activity, goal attempts, total throws, target shots and assists [19].

Therefore, this study aimed to evaluate 2017/2018 “Serie A” season data to compare the activity profiles and the position in the final ranking between the teams.

## Methods

### Data extraction

The data obtained by the Italian football league were anthropometric measurements and performance indicators of the matches.

### Experimental approach to the Problem

Through a detailed analysis of professional football teams during the 2017/2018 “Serie A” season, the importance of athletic performance and match statistics was assessed to reach the top positions in the final ranking.

The importance of athletic performance consists of the total distance covered in km, jogging, running and sprinting, and average speed. At the same time, match statistics include total shots, shots on the target, attempts, assists, turnovers and steals.

### Subjects

This analysis included the 20 teams of the “Serie A” championship. Data of all the teams registered for the 2017–2018 season of the “Serie A” were obtained from the Italian football federation’s statistics database.

### Procedures

The following parameters were collected from the official match reports of the Federazione Italiana Giuoco Calcio (www.legaseriea.it) for each match: total distance covered in km, jogging activity, running and sprinting activity, average speed, average distance covered per minute, total shots, shots on target, attempts on goal attempts, assists, turnovers, and steals. Based on speed ranges, three intensity zones were determined: walking (0-2.2 m/s), running (2.2–4.4 m/s), and sprinting (> 4.4 m/s). Performance activities and game results were also analysed according to how well they performed at the end of the season, dividing the population by final ranking position. Data were obtained in all the matches played during the 2017/2018 season for the Italian soccer federation using the same SICS system of multiple camera analysis (SICS, Bassano del Grappa, Italy) previously validated [20, 21].

### Statistical analysis

Statistical analysis was performed using SPSS (Chicago, IL) software for Macintosh (version 20.0). Values ​​are expressed as mean and standard deviation (SD). The Shapiro–Wilks test checked data normality. Since the normal distribution was assessed, the One-way ANOVA analysis was performed to test the mean differences of each variable (the percentage of jogging, running, sprinting, average speed, total goal, total shots, shots on target, goal attempts, assists, turnovers, steals and fouls suffered) between the teams. The pairwise comparisons to analyse the mean differences of each variable (the percentage of jogging, running, sprinting, average speed, total goal, total shots, shots on target, goal attempts, assists, turnovers, steals and fouls suffered) between the teams within the first four positions and teams ranked fifth and below with independent T-test were performed. The Pearson test (r) was used to estimate the correlation between total goals and athletic performance, total score or position in the final ranking.

The normality distribution was not assessed by dividing the data into the teams within the first four positions, and teams ranked fifth and below (the 1–4 positions group and 5–20 positions group). Therefore, to analyse the mean differences of each variable (total goal, total shots, shots on target, goal attempts, assists, turnovers, steals and fouls suffered) between the three phases of the season (1st-13th game, 14st-25th game and 26st-38th game) in the 1–4 positions and 5–20 positions groups the Kruskal-Wallis test was used.

A p-value of 0.05 was considered to indicate statistical significance.

## Results

### Athletic performance and the final ranking

Data from 380 games played by 20 teams participating in the “Serie A” championship during the 2017/2018 season were analysed. The average total distance covered by all the teams during the season was 109.24 ± 4.64 km, of which 25.36 % covered by jogging, 66.67 % running and 7.97 % sprint activities. The average speed was 6.78 ± 0.24 km/h.

The ANOVA analysis revealed that total distance covered, the percentage of jogging, running, sprinting, and average speed were significantly different (*p* < 0.001) among the teams. In particular, the teams within the first four positions (securing their places in the UEFA Champions League) revealed a statistically significant difference in the percentage of jogging (*p* = 0.037), running (*p* < 0.001) and sprinting (*p* < 0.001) compared to the other teams. Indeed, they showed a lower percentage of running (65.98 ± 1.51 vs. 66.84 ± 2.18; *p* < 0.001), a higher percentage of jogging (25.61 ± 1.71 vs. 25.30 ± 1.97; *p *= 0.037) and sprint activities (8.41 ± 1.04 vs. 7.86 ± 0.82; *p* < 0.001) compared to the teams ranked fifth and below. On the other hand, no statistically significant differences emerged for the total distance covered and average speed.

**Fig. 1:**
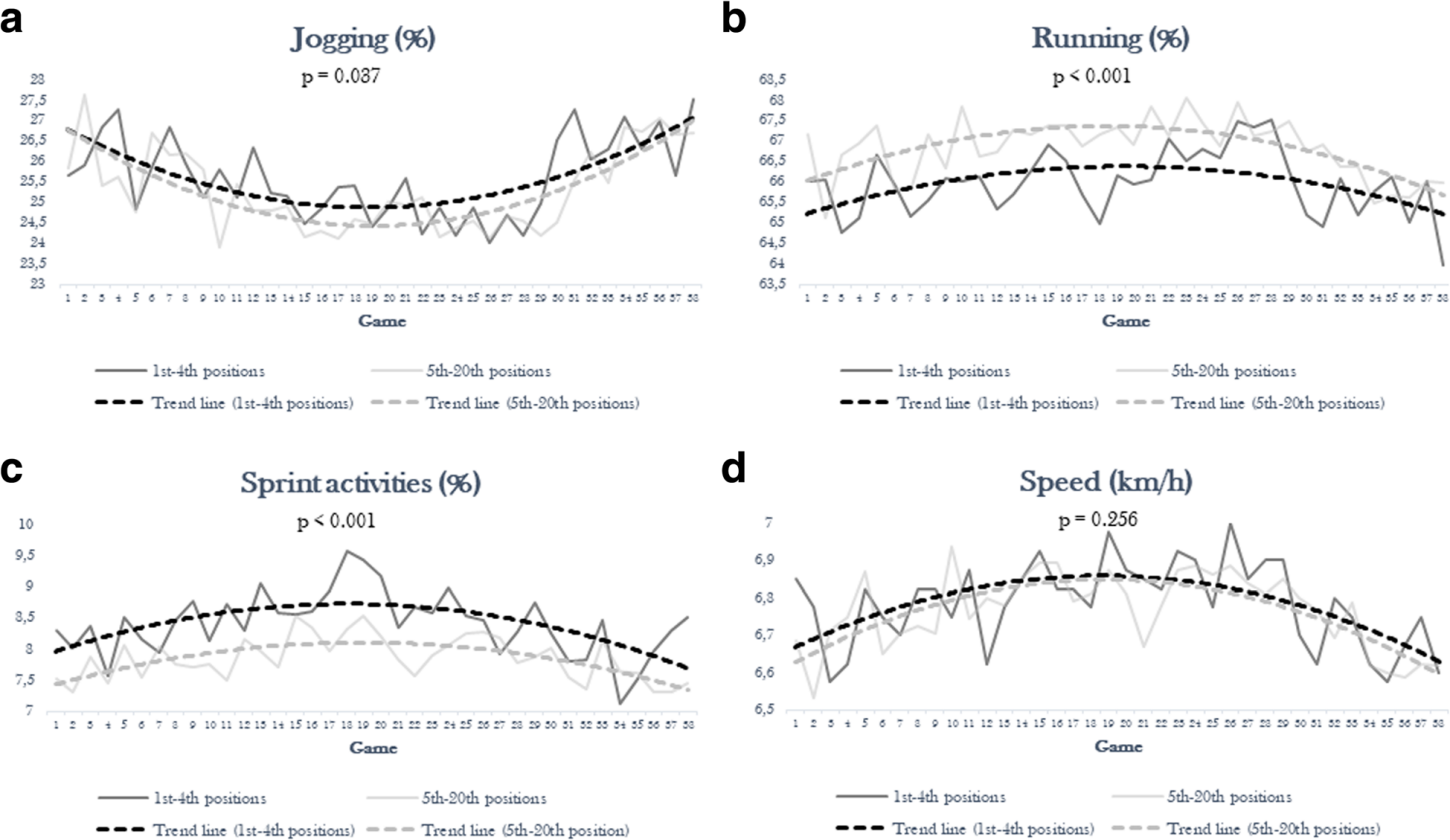
Percentage of jogging, running, sprint activities and average speed during the season 2017/2018 according to the final ranking (**a**: jogging; **b**: running; **c**: sprint activities; **d**: speed). Percentage of jogging, running, sprint activities and average speed during the season 2017/2018 according to the final ranking (a: jogging). Percentage of jogging, running, sprint activities and average speed during the season 2017/2018 according to the final ranking (b: running). Percentage of jogging, running, sprint activities and average speed during the season 2017/2018 according to the final ranking (c: sprint activities). Percentage of jogging, running, sprint activities and average speed during the season 2017/2018 according to the final ranking (d: speed )

To study the trend of athletic performance during the season, the average change in each parameter (for example, the percentage of jogging, running, sprint activities and average speed) for each game was traced and shown in Fig. 1 (a,b,c,d). The lowest jogging percentage was recorded on Day 10 and Day 26 for the lowest-ranked teams (23.8 %) and the top four teams (24 %). On the other hand, the highest percentage occurred on Day 1 (27.6 %) and Day 58 (27.5 %) for the two groups, respectively.

The lowest sprint activity was observed on Day 2 and Day 54 for the lowest-ranked teams (7.3 %) and the top four teams (7.1 %). On the other hand, the highest percentage was observed on Day 19 (8.2 %) and Day 18 (9.4 %) for the two groups.

The lowest average speed value was reached by the teams classified after fourth place on Day 2 (6.52 km/h) and by the first four teams on Day 55 (6.56 km/h). In contrast, the first group reached the highest average speed on Day 10 (6.91 km/h) and the second group on Day 26 (6.98 km/h).

The lowest running activity was observed on Day 2 and Day 58 for the lowest-ranked teams (65 %) and the top four teams (63.53 %). On the other hand, the highest percentage was observed on Day 24 (67.56 %) and Day 29 (67 %) for the two groups. Jogging percentage showed a downward trend in the first half of the season, while an upward trend appeared in the second half of the year, regardless of position in the final ranking. Conversely, running, sprint activities and average speed increased in the first half of the season and decreased in the second half.

### Match statistics and the final ranking

The correspondence statistics based on the final classification were examined through ANOVA one-way analysis. As shown in Table 1, total goals, total shots, shots on target, scoring attempts, assists, turnovers, steals, and fouls incurred appeared to be statistically different (p < 0.001) between teams. Besides, similar differences emerged when comparing the top four teams to the other lower-ranked teams.

**Table 1:** Match statistics according to the final ranking

Rank	Total goals, n	Total shots, n	Shots on target, n	Goal attempts, n	Assists, n	Turnovers, n	Steals, n	Fouls suffered, n
1st	2.26 ± 1.55	11.55 ± 4.67	7.08 ± 3.07	8.11 ± 3.65	3.47 ± 1.81	37.05 ± 8.25	26.53 ± 6.94	12.76 ± 4.67
2nd	2.03 ± 1.50	14.42 ± 4.85	8.34 ± 3.99	9.79 ± 3.69	4.27 ± 2.21	42.47 ± 8.67	29.00 ± 6.32	9.71 ± 3.46
3rd	1.63 ± 1.36	13.39 ± 5.09	7.21 ± 3.43	9.29 ± 3.88	3.70 ± 2.40	38.16 ± 6.77	31.03 ± 7.71	11.16 ± 3.94
4th	1.68 ± 1.42	12.66 ± 5.36	7.32 ± 3.68	9.18 ± 3.99	3.75 ± 2.31	36.58 ± 8.07	29.24 ± 7.05	10.63 ± 3.14
5th	2.34 ± 1.67	12.37 ± 5.48	7.24 ± 3.68	9.13 ± 4.04	3.53 ± 2.46	36.47 ± 8.42	29.68 ± 8.07	9.47 ± 3.07
6th	1.50 ± 1.31	13.21 ± 4.65	7.97 ± 3.37	8.24 ± 3.25	3.41 ± 2.00	35.00 ± 6.80	26.21 ± 6.74	12.92 ± 5.18
7th	1.47 ± 1.25	12.21 ± 5.16	7.21 ± 3.35	9.11 ± 3.91	3.44 ± 1.78	37.08 ± 8.61	30.32 ± 8.07	12.34 ± 3.79
8th	1.42 ± 1.15	12.97 ± 5.30	6.61 ± 2.78	8.19 ± 4.35	3.14 ± 1.96	32.08 ± 7.60	28.89 ± 8.49	10.92 ± 3.64
9th	1.37 ± 1.17	9.84 ± 3.98	5.39 ± 2.57	7.50 ± 3.67	3.06 ± 1.65	37.63 ± 10.06	27.71 ± 6.94	14.05 ± 3.74
10th	1.47 ± 1.29	10.18 ± 3.77	5.97 ± 2.98	6.76 ± 2.49	3.36 ± 2.16	36.42 ± 9.41	26.37 ± 7.17	12.66 ± 3.75
11th	0.79 ± 0.66	10.13 ± 2.99	5.21 ± 2.21	6.32 ± 2.41	2.34 ± 1.47	35.32 ± 8.36	28.21 ± 7.41	14.84 ± 4.13
12th	0.82 ± 0.90	8.95 ± 4.79	4.65 ± 2.56	5.95 ± 2.86	2.82 ± 1.59	35.76 ± 8.71	28.58 ± 9.56	12.79 ± 4.62
13th	0.97 ± 0.85	8.18 ± 3.52	4.41 ± 2.56	5.08 ± 2.83	2.26 ± 1.77	31.42 ± 7.88	25.97 ± 5.57	11.87 ± 4.97
14th	1.32 ± 1.07	9.45 ± 3.73	4.58 ± 2.51	5.89 ± 3.48	2.62 ± 1.57	33.87 ± 8.30	25.84 ± 5.92	10.32 ± 2.87
15th	1.05 ± 0.93	7.74 ± 2.77	4.68 ± 2.04	5.82 ± 2.47	2.15 ± 1.18	34.97 ± 6.74	25.50 ± 5.72	12.21 ± 3.27
16th	0.84 ± 0.79	7.65 ± 3.42	4.25 ± 2.22	5.44 ± 2.90	2.45 ± 1.38	31.03 ± 7.25	25.39 ± 7.53	11.82 ± 3.53
17th	0.97 ± 0.92	7.66 ± 3.72	4.36 ± 2.47	5.49 ± 2.95	2.15 ± 1.40	33.79 ± 7.54	24.97 ± 6.82	16.37 ± 4.53
18th	0.95 ± 1.11	8.39 ± 3.40	4.45 ± 2.04	5.29 ± 2.98	2.39 ± 1.91	33.32 ± 8.84	27.03 ± 6.58	12.63 ± 4.58
19th	0.71 ± 0.84	6.87 ± 3.34	3.63 ± 2.16	4.56 ± 2.59	2.08 ± 1.12	35.08 ± 10.66	27.08 ± 8.07	10.74 ± 3.39
20th	0.87 ± 1.02	9.68 ± 4.34	5.19 ± 3.21	6.55 ± 2.96	2.30 ± 1.21	33.24 ± 7.77	25.61 ± 8.34	12.26 ± 4.19
***p*-value**	< 0.001	< 0.001	< 0.001	< 0.001	< 0.001	< 0.001	< 0.001	< 0.001

Data are expressed as mean ± SD

One-way univariate ANOVA analysis

As expected, the Pearson correlation analysis revealed that at the end of the championship, the total goals are strongly associated both with the total score (*R* = 0.906; *p* < 0.001) and with the position in the final ranking (*R* = 0.850; *p* < 0.001). Also, a statistically but not clinically significant positive correlation was demonstrated between total goals and total distance covered (*R* = 0.081; *p* = 0.026), sprint activities (*R* = 0.197; *p* < 0.001), and average speed (*R* = 0.101; *p* = 0.005). On the other hand, a statistically and clinically significant negative correlation was demonstrated between total goals and running (*R*=-0.72; *p* = 0.046). Therefore, as the number of goals increased, running activity decreased.

To find any differences in match statistics based on the season phase, all games were divided into tertiles and analysed separately (Table 2). Total shots and goal attempts showed a downward trend during the season (*p* = 0.024 and *p* < 0.001, respectively). Furthermore, turnovers and steals seemed to be higher in the second tertile (*p* = 0.001). Simultaneously, shots on targets were lower in the second tertile, with a statistically significant difference, referring only to the first four positions (*p* = 0.027).

**Table 2:** Match statistics according to the tertiles of championship

	1st -13th game	14st -25th game	26st -38th game	p for trend
**Total goal**, n				
All	1.46 ± 1.33	1.26 ± 1.19	1.30 ± 1.26	0.247
1st -4th positions	2.33 ± 1.42	1.68 ± 1.49	1.75 ± 1.45	0.027
5st -20th positions	1.24 ± 1.21	1.16 ± 1.08	1.19 ± 1.18	0.885
**Total shots**, n				
All	10.86 ± 4.39	10.36 ± 5.00	9.92 ± 4.79	0.024
1st -4th positions	13.83 ± 4.46	12.88 ± 5.35	12.33 ± 5.25	0.256
5st -20th positions	10.11 ± 4.06	9.74 ± 4.71	9.31 ± 4.48	0.057
**Shots on target**, n				
All	6.03 ± 3.17	5.80 ± 3.10	5.59 ± 3.34	0.142
1st -4th positions	8.35 ± 3.31	7.07 ± 3.40	7.11 ± 3.90	0.051
5st -20th positions	5.44 ± 2.86	5.47 ± 2.94	5.20 ± 3.08	0.377
**Goal attempts**, n				
All	8.18 ± 3.66	6.81 ± 3.57	6.35 ± 3.52	< 0.001
1st -4th positions	10.98 ± 3.37	8.59 ± 3.67	7.79 ± 3.74	< 0.001
5st -20th positions	7.47 ± 3.38	6.36 ± 3.41	5.98 ± 3.38	< 0.001
**Assist**, n				
All	3.17 ± 1.97	2.89 ± 1.86	2.87 ± 1.94	0.120
1st -4th positions	4.38 ± 2.35	3.61 ± 2.20	3.42 ± 1.93	0.096
5st -20th positions	2.84 ± 1.71	2.68 ± 1.70	2.71 ±1.92	0.435
**Turnovers**, n				
All	35.58 ± 7.88	36.44 ± 8.82	33.80 ± 8.81	0.001
1st -4th positions	38.35 ± 8.14	39.50 ± 7.83	37.69 ± 8.82	0.372
5st -20th positions	34.89 ± 7.67	35.68 ± 8.90	32.83 ± 8.56	0.002
**Steals**, n				
All	27.09 ± 7.21	28.78 ± 7.51	26.29 ± 7.37	0.001
1st -4th positions	29.52 ± 7.43	28.98 ± 6.37	28.33 ± 7.76	0.660
5st -20th positions	26.48 ± 7.04	28.73 ± 7.78	25.78 ± 7.20	< 0.001
**Fouls suffered**, n				
All	12.03 ± 4.18	12.47 ± 4.34	11.82 ± 4.23	0.106
1st -4th positions	11.10 ± 4.16	11.09 ± 4.11	11.00 ± 3.70	0.980
5st -20th positions	12.26 ± 4.16	12.81 ± 4.34	12.03 ± 4.33	0.077

Data are expressed as mean ± SD

Kruskal-Wallis test for comparisons among groups

## Discussion

After the Spanish Liga and the English Premier League, Italian Serie A ranks third in the UEFA standings as the highest-level competition. At the end of the 38 championship days, the first four classified teams are admitted to the Champions League, the fifth and sixth-ranked teams secure their places in the UEFA Champions League, and the last three teams in the final ranking relegate to Serie B, replaced by as many teams promoted by the cadet championship.

Because of this intricate interweaving between the various national, European and world championships, our study focused on the differences between the 20 teams to understand which athletic performance characteristics may advance in the final ranking. The previous analysis of the 2016/2017 Serie A season showed significant differences between the teams in jogging, running, and sprinting [19]. The present study highlighted an association between the total distance covered and the highest positions in the final ranking. Moreover, the teams within the first four positions showed a lower percentage of running activity, a higher rate of jogging and sprint activities than the teams ranked fifth and below.

Soccer is defined as an intermittent sport because high-level players’ physical demands are mainly irregular racing actions. In the last decade, many studies have documented the physical performance of soccer players demonstrating that an athlete covers, on average, about 11 km during a game [22–25]. However, due to the game’s intermittent nature, the total distance covered cannot be an adequate parameter for understanding the overall physical requirements. Therefore, the distance covered at very high speed seems to be a better indicator of performance than the total distance covered [7, 26–28]. For this reason, we also investigated the average speed between the 20 teams, which was increased in the teams ranked in the top positions.

Due to the increase in average speed and the total distance covered by the teams ranked in the highest positions, we have performed an in-depth analysis between the top four ranked teams (who can participate in the Champions League) and the remaining teams. It has been found that the first four teams have a higher percentage of jogging and sprint activities and lower running activities than the other teams. On the other hand, no statistically significant difference was found between the two groups in terms of average speed and total distance covered. These findings agree with another study on the English premier league, which showed that the best teams in the final ranking had a lower percentage of running than the other lower-level teams during the game [27]. However, these results, while statistically significant, have a faint clinical correlation. Therefore, future studies that monitor athletes’ activities are needed to investigate how these variables may influence the outcome to target individual athletes’ training.

Several studies have shown that players, but also referees, who completed the most significant amount of physical activity, in the first half experienced decreased physical performance in the second half [1, 15, 29]. It has been demonstrated that the decline in physical work is independent of the final rank, and it is linked to physical fatigue, which increases with a higher percentage of running and sprinting [12, 28, 30, 31]. Therefore, we can speculate that the lower running rate may be a tactical choice to save physical load and achieve better results. Thus, these findings could justify the higher percentage of jogging in the best teams. Still, also, they might explain the general trend during the season of jogging, average speed, running and sprint activities. The present study analysed the teams’ trends during the first and the second half of the 2017/2018 Serie A season, highlighting the average change in each GPS-derived metrics for each game. Our results showed a downward trend in jogging in the first half of the season, while an upward trend appeared in the second half of the year. On the other hand, conversely to the second half, an increase in running activity time, sprint activity, and average speed occurred in the first half of the season.

Little is known about the potential correlations between the team’s success, the technical performance intended as the total number of short passes and the number of successful short passes [12]. Therefore, to highlight any differences between the teams in terms of match statistics based on the final ranking, we studied the potential association of total goals, shots on the target, goal attempts, assists, turnovers, and steals with the highest positions. The Serie A championship scoring system awards 3 points for the victory, one point for each team for the tie and no points for the defeat. In the event of a tie, the following parameters are considered for the formulation of the final ranking: the competitions between the formations concerned, the goal difference of the direct matches, the overall goal difference, the highest number of goals scored. In agreement with the literature results, the first four teams had more total throws, shots on goal, goal shots, assists and turnovers than the other lower-ranked teams [12]. The total number of goals appeared to be positively correlated with sprint activities and negatively related to the running activity duration. Notably, these athletic performance characteristics are typically found in teams ranked in the highest positions in the final ranking. These significant differences can help identify a winning profile, especially as these results are similar to those of the previous 2016/2017 Serie A season study.

To elucidate whether these differences in match statistics may be affected by the season phase, we divided and analysed all games separately into tertiles. Total shots and goal attempts showed a downward trend during the season. An upward trend of turnovers and steals and a downward trend of total shots on target appeared, with a statistically significant difference referring only to the first four teams. As for athletic performance, match statistics showed a different trend during the season, suggesting that both athletic performance and game variables are critical factors for victory.

## Conclusions

These data provide valuable information on athletes and teams’ performance profile to reach the highest positions in the Serie A season’s final rankings. We can speculate that teams in the highest ranks of the final standings should have professional soccer training comprising a lower percentage of running and a higher percentage of jogging and sprinting than other teams. Furthermore, this athletic performance profile seems to be related to the higher achievement of goals, total shots, shots on target, attempts, assists, turnovers, steals and fouls suffered.

Most of the previous studies of game analysis examined only the technical and tactical performance of the player. However, due to the multi-skilled nature of soccer, further research should investigate the role of psychology in athletes’ performance and whether it could influence the team’s success in reaching the top of the rankings.

## Data Availability

The datasets generated and/or analysed during the current study are available from the corresponding author on reasonable request.

## Abbreviations

SD: Standard deviations
ORS: Odds ratios
CI: Confidence intervals
